# Acquired resistance for immune checkpoint inhibitors in cancer immunotherapy: challenges and prospects

**DOI:** 10.18632/aging.203833

**Published:** 2022-01-17

**Authors:** Xunrui Chen, Wenhui Zhang, Wenyan Yang, Min Zhou, Feng Liu

**Affiliations:** 1 Department of Oncology, Shanghai Ninth People’s Hospital, Shanghai Jiao Tong University School of Medicine, Shanghai 201900, China; 2 Shanghai Institute of Precision Medicine, Shanghai 200125, China; 3 Medical Center, Shanghai Ninth People's Hospital, Shanghai Jiaotong University School of Medicine, Shanghai 201900, China; 4 Department of Respirtory Medicine, Jinshan Branch of the Sixth People’s Hospital of Shanghai, Shanghai 201599, P.R. China

**Keywords:** immunotherapy, acquired resistance, immune checkpoint inhibitor, the tumor microenvironment, combination therapy

## Abstract

Drug resistance has become an obstacle to the further development of immunotherapy in clinical applications and experimental studies. In the current review, the acquired resistance to immunotherapy was examined. The mechanisms of acquired resistance were based on three aspects as follows: The change of the tumor functions, the upregulated expression of inhibitory immune checkpoint proteins, and the effects of the tumor microenvironment. The combined use of immunotherapy and other therapies is performed to delay acquired resistance. A comprehensive understanding of acquired drug resistance may provide ideas for solving this dilemma.

## INTRODUCTION

Tumorigenesis and immunity have been extensively studied, and immunotherapy against cancer is undoubtedly becoming a research hotspot. This method is mainly divided into the two following categories: Immune checkpoint inhibitors (ICIs) and adoptive cell transfer (ACT) therapy. At present, ICIs are more widely used. ICIs enhance the antitumor immune response of patients by blocking immune checkpoints (ICs) that inhibit the immune function of the body [1]. Anti-PD-1/PD-L1 antibodies are considered one of the most famous ICIs. A review estimated the objective response rate for patients who treated with Pembrolizumab in melanoma, non-small cell lung cancer (NSCLC), renal cell carcinoma (RCC), Hodgkin lymphoma, urothelial carcinoma, esophageal cancer, and head and neck squamous cell carcinoma were 52%, 42%, 36%, 72%, 29%, 10%, and 17%, respectively [2]. In addition, certain responders will become non-responders following treatment with ICIs. For example, 20% of responders with reactive melanoma who were treated with anti-PD-1 inhibitors achieved a complete response (CR), and 55% of them achieved a partial response (PR), and subsequently developed acquired resistance [3].

The antitumor immune response can be regarded as a tumor-immune cycle, which is characterized by the following processes: 1) Release of antigens by tumor cells; 2) antigen presentation; 3) immune response activation; 4) T cell transportation to the tumor site; 5) T cell infiltration in the tumor site; 6) T cell antigen recognition, and 7) tumor cell killing, and release of antigens [4]. Acquired resistance can interfere with the antitumor immune response from the first step. Certain factors affect the antitumor immune response of the immune system. The stronger the immunogenicity of the tumor neoantigen, the more potent the immune response [5]. MHC-I and MHC-II restricted neoantigens play crucial roles in the antitumor response [6, 7]. Programmed death-ligand 1 (PD-L1), microsatellite instability/defective mismatch repair (MSI/dMMR), and tumor mutational burden (TMB) have been regarded as biomarkers that can predict the efficacy of immunotherapy [8]. The mining of tumor database also reveals other factors, such as hormones affecting the efficacy of ICIs [9], and has guiding significance for the use of ICIs, such as for head and neck squamous cell carcinoma [10].

Depending on the different mechanisms and time of response, drug resistance induced by ICIs can be divided into primary resistance and acquired resistance.

Acquired resistance is noted in patients who experience a temporary, partial, or complete clinical response following immunotherapy, which is ultimately followed by clinical and/or radiological progression of the disease [2, 11, 12]. In essence, both tumor cells and the tumor microenvironment (TME) are modified following their interaction with the immune system. New characteristics are obtained that prevent the tumor from being attacked by the immune cells [13]. The mechanisms can be interpreted by changes occurring in the tumor functions, changes in the expression levels of the inhibitory checkpoint proteins, and changes in the TME components.

Primary drug resistance is mainly caused due to somatic DNA aberrations, which influence the expression levels of tumor-associated antigens and the quantity and quality of immune effector cells in the TME. For example, the activation, migration, and infiltration of T cells into the TME are blocked. These changes result in patients not responding to the initial immunotherapy, which in turn leads to rapid disease progression [2, 14, 15].

Certain non-responders have been reported that are often confused with subjects developing primary resistance and acquired resistance. Adaptive resistance is one of these types of resistance. It refers to tumor cells that are successfully identified by the immune system at the beginning, but are gradually adapted to the pressure of the immune attack to develop resistance [13]. Two conditions have to be met to acquire adaptive resistance. Firstly, gradual development is noted in the processes of tumor growth and the interaction between the tumor and the immune response, and secondly, the mechanism is limited only to the TME [11]. This response can be clinically expressed as primary resistance, acquired resistance, and mixed resistance [13].

An alternative scenario includes immune escape, by which ICIs exhibit an initial therapeutic effect and drug-resistant tumor cell clones that exist inherently are subsequently selected, proliferate, and eventually develop resistance [13]. The mechanism of action is classified as intrinsic resistance. Due to the presence of these clones that lack the drug targets, this type of resistance should not be regarded as actual drug resistance [11].

**Figure 1:**
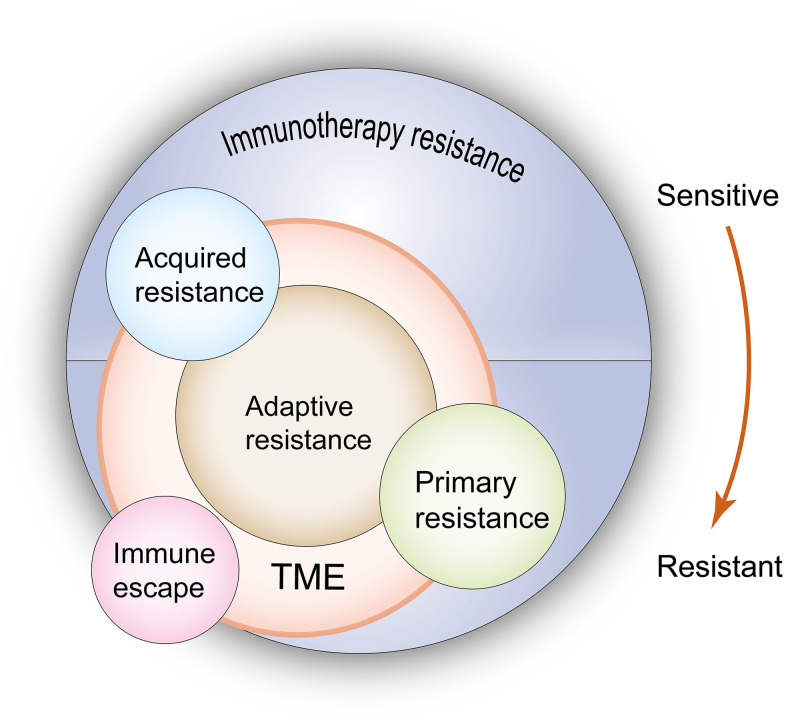
**Relationship between primary resistance, acquired resistance, adaptive resistance and immuno-escape when undergoing immunotherapy.** According to the resistance mechanisms, primary and acquired resistance result from the changes of both tumor cells and the TME, but adaptive resistance is only limited to the latter. In terms of clinical features, adaptive resistance shows the same characteristics as primary resistance or acquired resistance. Immune escape leads to the same outcome as drug resistance, but due to these clones lack drug targets, this type of resistance should not be regarded as a real drug resistance. TME: tumor microenvironment. The red arrow indicates the evolution of immune state from sensitivity to drug resistance, which corresponds to the state of acquired resistance and primary resistance when immunotherapy is used.

The relationship of these confusing concepts in ICI resistance is shown in Figure 1. Distinguishing the aforementioned conceptions contributes to a better understanding of the theme of this review, which examines acquired resistance. Understanding the mechanisms of acquired resistance following treatment with ICIs is helpful to explore ways to overcome this challenge.

## THE ANTECEDENTS AND CONSEQUENCES OF ACQUIRED RESISTANCE FOR ICIS

**Figure 2:**
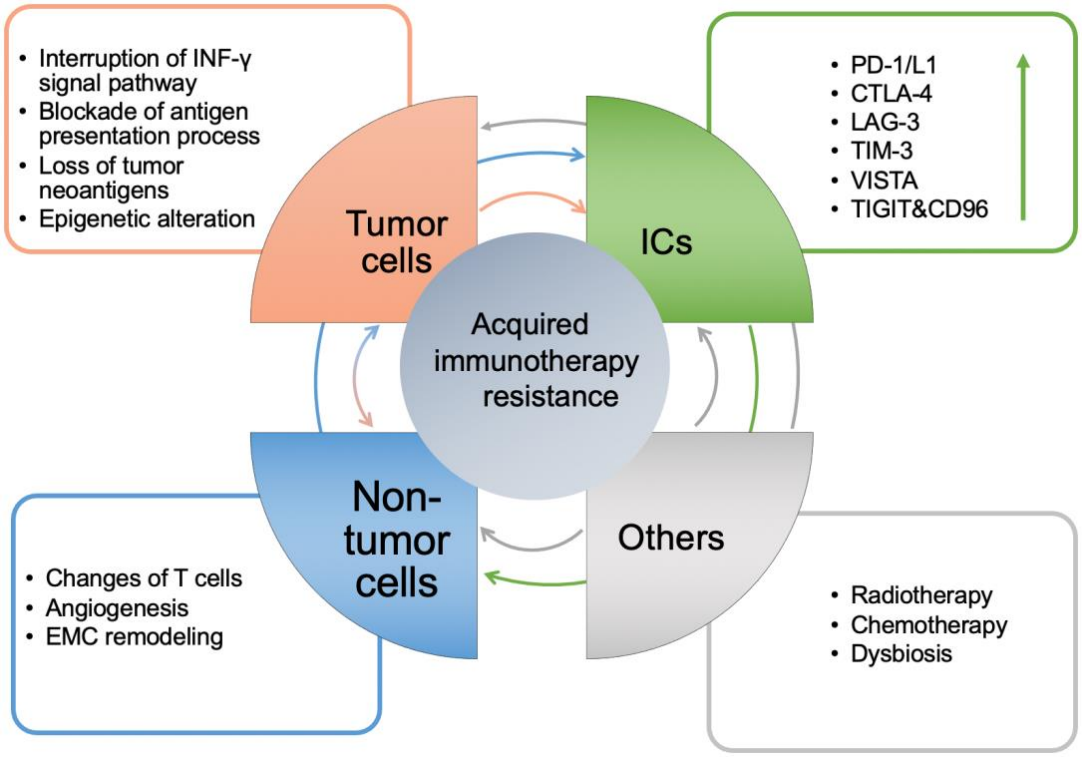
**The mechanisms of acquired resistance of immunotherapy and the interaction between them.** Generally, it is manifested in four aspects: tumor cell itself, the level of ICs, non-tumor cells in the TME and others. The arrow between modules represents the direction of regulation, and its color corresponds to each module. The arrow in the ICs module represents up regulation.

The occurrence of acquired resistance following the use of ICIs can be summarized into the three following mechanisms: Mutation of tumor cells, upregulation of inhibitory ICs, and increase in the immunosuppressive components of the TME. These mechanisms interact with each other as shown in Figure 2, finally leading to patient acquired resistance.

## Tumor-mediated acquired resistance

### Gene mutations in tumor cells

### Interruption of the IFN-γ signaling pathway

IFN-γ plays a paradoxical role in regulating antitumor immunity. Initially, it increases the sensitivity of cancer cells to the induction of the apoptotic pathways, while interfering with the proliferation and survival of endothelial cells in the TME. In addition, it hinders angiogenesis and enhances the expression levels of MHC on antigen presentation cells (APCs) [16]. It has been clinically used to treat certain malignant tumors as an immunomodulatory agent [17]. In contrast to these observations, IFN-γ has the potential to induce tumor progression. For example, IFN-γ is secreted by tumor-associated macrophages (TAMs), which can upregulate PD-L1 expression in lung cancer cells [17]. The inhibition of the IFN-γ signaling pathway is more prone to cause damage to the antitumor immune response. A classic example regarding the interruption of the IFN-γ signaling pathway is the loss-of-function mutation of the JAK1/2 in tumor cells, notably of JAK2, which was detected in recurrent patients with melanoma treated with anti-PD1/L1 [18]. However, the sequencing of the barcoded shRNAs revealed that Ntrk1 expression was upregulated in tumors treated with PD-1 inhibitors, which regulate Jak/Stat signaling to promote expression of PD-L1 in tumor cells and cause CD8+ T cell exhaustion [19]. These results illustrate that the imbalance of the IFN-γ signaling pathway caused by ICI treatment contributes to the development of acquired resistance.

### Blockade of the antigen presentation process

Beta-2-microglobulin (β-2M) is a common component of MHC-I that presents antigens to CD8+T cells [18]. The truncated mutation of the β-2M gene leads to the failure of the tumor cells to be recognized by tumor-specific CD8+T cells [20]. Downregulation or loss of β-2M expression was detected in patients with lung cancer or melanoma who had been treated with ICI therapy and subsequently developed acquired resistance [18, 21]. In addition, HLA-A, -B, and -C mutations indicated a positive association with effector lymphocyte activity, suggesting that these mutations were caused by the immune attack and that they could subsequently block the antigen presentation process [22, 23].

### Loss of tumor neoantigens

Loss of neoantigens associated with mutations was found in tumor patients who had developed acquired resistance following immunotherapy [24]. The so-called neoantigens refer to the molecules that bind to MHC with high affinity or affect TCR contact and exhibit high immunogenicity [25]. Two mechanisms of neoantigen loss have been reported in acquired resistant tumors as follows: 1) Tumor cells containing neoantigens are eliminated by immunity, and subsequently the remaining tumor cells proliferate, and 2) the tumor cells obtain one or more genetic events, such as HLA mutation, which result in loss of neoantigens and selection and expansion of resistant clones [24]. Loss of new tumor antigens was also found in patients with NSCLC treated with ICIs [24, 26]. Loss of CD19, which was expressed in the majority of B-cell acute lymphoblastic leukemia (B-ALL), was detected in relapses following chimeric antigen receptor T-cell immunotherapy (CAR-T) treatment of B-ALL patients [27].

In addition, a previous study reported that activation of the β-catenin signaling pathway or PTEN gene deletion were two oncogenic aberrations linked to ineffective T cell infiltration into tumor sites that promoted acquired resistance for patients with metastatic melanoma following combination with anti-CTLA-4 and anti-PD-1 therapy [28].

### Epigenetic alteration in tumor cells

This term is used to describe the molecular pathway that regulates gene expression without altering the DNA sequence. It has become a key area of cancer development and progression. The mechanisms of epigenetic alteration include DNA methylation and histone enzyme modification [29]. This affects the expression levels and presentation of tumor antigens, the functions of T cells including CD8+T and regulatory T cells (Tregs), and the abundance of myeloid-derived suppressor cells (MDSCs) and TAMs [30]. Certain studies have shown that in patients with colon cancer, tumor cells can evade cell lysis and cause an upregulation of the expression levels of immune checkpoint proteins, such as PD-1, CTLA-4, TIM-3, TIGIT, PD-L1, and galectin-9 by downregulating DNA methylation and repressing histone modification. These epigenetic modifications can be used as biomarkers for the diagnosis of colorectal cancer (CRC) [31].

## Compensatory upregulation of the expression levels of inhibitory ICs used in immunotherapy

Although the uses of anti-PD-1/L1 and anti-CTLA-4 antibodies have been approved by the U.S. Food and Drug Administration (FDA), it has been shown that LAG-3, TIM-3, TIGIT, VISTA have the potential to be upregulated in patients with tumor recurrence following anti-PD-1/L1 or anti-CTLA-4, which implies another mechanism for the acquired resistance.

### LAG-3: Lymphocyte activator gene 3, CD223

Following RNA-sequencing analysis and IHC on a series of anti–PD-1–treated melanoma and non–small cell lung cancers, LAG-3 expression was upregulated. LAG-3 is exclusively expressed by T cells, which bind to MHC II with a higher affinity than that of CD4+T cells that suppress MHC-II–mediated antigen presentation and antigen-specific CD4+ effector T cell reaction and cytokine production [32]. LAG-3 promotes Treg differentiation and impedes the differentiation of monocytes into macrophages and dendritic cells (DCs), which in turn weaken the immunostimulatory ability [33]. In a mouse ovarian cancer model treated with anti-CTLA-4 or anti-PD-1, the expression levels of LAG-3 on CD8+ T cells were increased [34].

### TIM-3: T cell immunoglobulin 3, CD366

Similar to LAG-3, TIM-3 expression is upregulated following treatment of melanoma and non–small cell lung cancers with anti–PD-1 antibodies, which may be one of the reasons for the development of acquired resistance [32]. TIM-3 is a transmembrane protein that can be expressed in CD4+TH1 and CD8+ cytotoxic T cells, Treg cells, DCs, NK cells, monocytes, and macrophages [35]. The three following ligands are combined with TIM-3 that regulate antitumor immunity: Galactose lectin-9 (Galectin 9), phosphatidylserine (PtdSer), and high mobility group box 1 (HMGB1). The combination of galectin 9 and TIM-3 can negatively regulate the Th1 immune response by inducing T cell apoptosis. In the anti-PD-1 resistant Kras mouse model loaded with lung adenocarcinoma tumors, the expression levels of RNA and proteins encoding Galectin 9 were higher than those noted in the model without anti-PD-1 treatment [36]. A previous study that examined TIM-3 expression in lung cancer cells *in vitro* indicated that the increased expression of this protein on CD8+ T cells led to the decrease of IFN-γ levels [37]. Compared with TIM-3-Treg, TIM-3+ Treg released more IL-10 in melanoma and colon cancer mouse models, indicating enhanced immunosuppressive activity [38].

### VISTA: V-domain Ig inhibitor activated by T cells

The upregulation of VISTA expression in melanoma patients treated with anti-PD-1 suggests that it may play an important role in acquired drug resistance [39]. VISTA, also known as PD-1 homolog (PD1H), belongs to the B7 family of proteins. It encodes type I membrane proteins, which are mainly expressed in hematopoietic cells, such as myeloid cells, granulocytes, and T cells [40]. Furthermore, it negatively regulates the CD4+ T cell response by inhibiting early TCR activation and arresting the cell cycle [41]. It also inhibits the production of IFN-γ and IL-2 in CD8+ T cells. The latter is significant for the survival and proliferation of T cells [40]. In the mouse model, the number and activity of effector T cells (Teffs) was increased by blocking VISTA expression, while the infiltration of Treg and MDSC cells was decreased [42].

### TIGIT: T cell immunoglobulin and ITIM domain protein

In several tumors, such as melanoma, the expression levels of TIGIT in CD8+ T cells are upregulated, and the high TIGIT/DNAM1 ratio in Tregs is associated with poor prognosis following PD-1 and/or CTLA4 pathway blockade [43, 44]. TIGIT is a co-inhibitory receptor expressed on lymphocytes and belongs to the poliovirus receptor vPVR/nectin family [45]. It has a higher affinity for CD155-CD112 and competes with CD226. Its binding disrupts the activation of costimulatory signal transduction pathways [46]. Particularly, TIGIT plays a role in regulating antitumor immunity mediated by tumor-infiltrating microorganisms. A previous study has shown that the abundance of *Clostridium sclerotium* in colorectal cancer with high TIGIT expression is significantly increased, which is related to poor prognosis and high recurrence rate following treatment with ICIs [47].

## The changes of the TME contribute to acquired resistance

**Figure 3:**
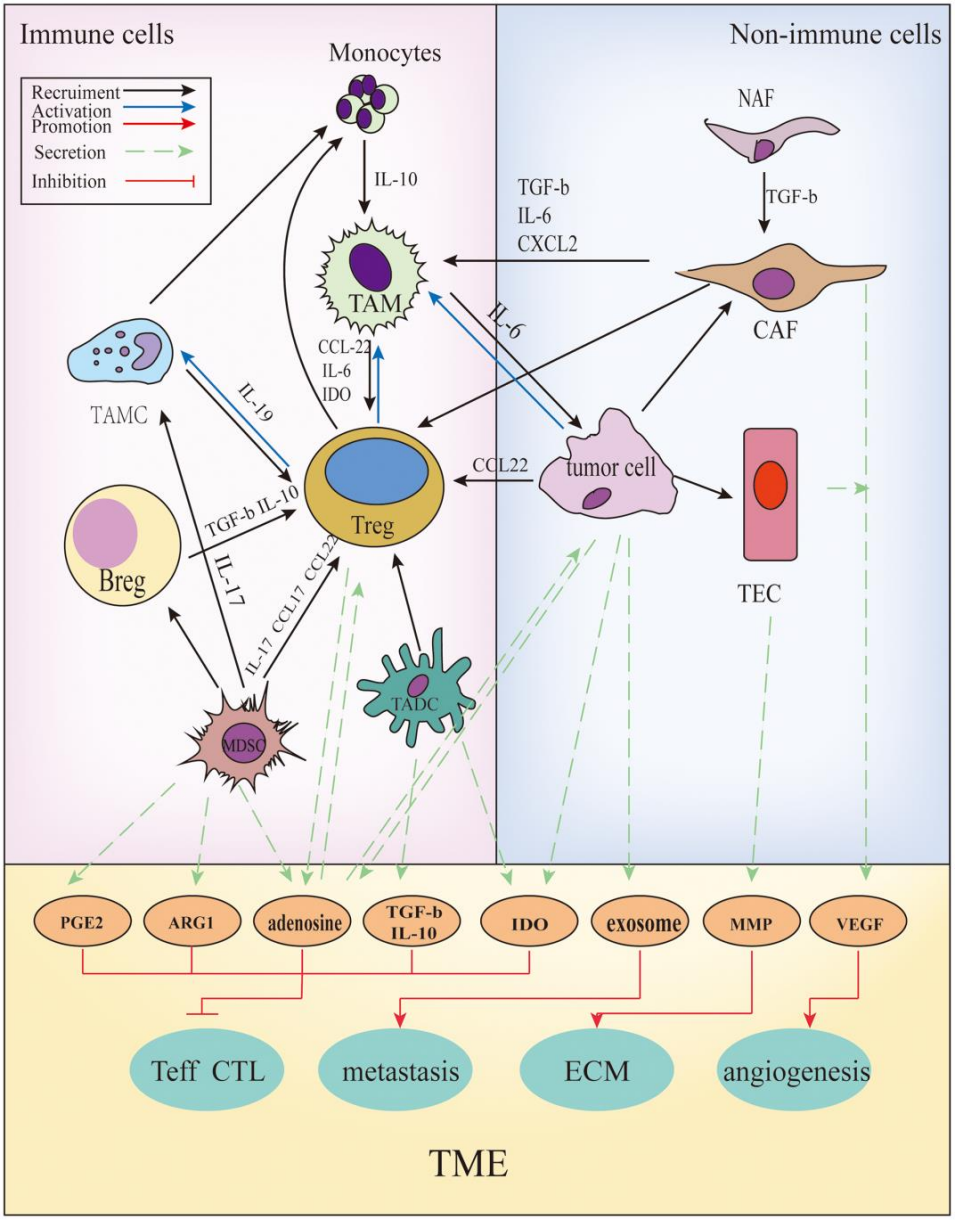
**The cross-talks between immune and non-immune cells within the TME.** In the TME, all kinds of cells secrete soluble molecules and interact with each other, such as prostaglandin E2(PGE2), arginase-1(ARG1), adenosine, transforming growth factor β(TGF-β), interleukin (IL)-10, indoleamine2,3-dioxygenase (IDO), exosomes, matrix metalloproteinase (MMPs), vascular endothelial growth factor (VEGF), and so on. These factors inhibit the function and proliferation of CD4+/ CD8+ T cells, promote angiogenesis, extracellular matrix (ECM) remodeling and tumor metastasis.

Cancer cells can functionally sculpt their microenvironment through the secretion of various cytokines, chemokines, and other factors [48]. The TME is conducive to genes and epigenetic changes caused in the tumor. In addition to tumor cells, immune cells are present in the TME, such as T cells, B cells, tumor-associated macrophages (TAMs), tumor-associated dendritic cells (TADCs), tumor-associated mast cells (TAMCs), and non-immune cells. These include tumor-associated endothelial cells (TAEs) and cancer-associated fibrous cells (CAFs). In addition, blood vessels and the extracellular matrix (ECM) (including collagen, matrix metalloprotein kinase) components are present [49]. The regulation between various components is shown in Figure 3. Acquired resistance is induced by specific changes that are discussed in the following four sections.

### Increase of suppressor T cells and decrease of effector T cells

Upregulation of the expression of specific IC proteins following anti-PD-1/CTLA4, such as TIM-3 causes an increase in TGF-β and IL-10 secretion, which in turn promotes the differentiation and expansion of Tregs and the induction of MDSCs. Tregs upregulate the expression of indoleamine 2,3-dioxygenase (IDO) in DCs through the interaction of CTLA-4 and B7 ligands, and IDO+ DCs induce the conversion of Teffs into Tregs [50]. MDSCs can reduce the ratio of CD8+ T to Treg cells, and the released Prostaglandin E2 (PGE2), arginase 1 (ARG-1), and inducible Nitric oxide synthase (iNOS) can inhibit the cell cycle of T cells and participate in tumor aggregation [51]. TAMs produce chemokines and recruit Th2 cells and Tregs [52]. Sustained type I interferon signaling following therapy with anti-PD-1 monoclonal antibodies (mAb) induces NOS expression in both tumor and dendritic cells (DCs), which is associated with intratumor accumulation of Tregs and myeloid cells and acquired resistance [53]. These aggregated inhibitory immune cells affect the differentiation and activation of effector T cells. Treg cell apoptosis induces activation of adenosine during hypoxia, which inhibits the activation and proliferation of Teff and the release of cytokines from CD4+/CD8+T cells [54]. Regulatory B cells (Bregs) inhibit the activity of Teff by releasing anti-inflammatory cytokines, such as IL-10 and IL-35 [55]. MDSCs inhibit the cytotoxicity of CD8+ T cells in an antigen-specific manner [51]. TADCs release IL-10 and TGF-β to inhibit the activation of cytotoxic CD8+ T cells [56]. CAF promotes apoptosis of CD8+ T cells [57].

### Up-regulation of ICs

TAMs and Bregs express co-suppressor molecules, such as PD-L1 [58, 59]. Tregs can upregulate the expression levels of PD-1, CTLA-4, LAG-3, TIM-3, TIGIT, and VISTA [60, 61]. TADCs can express TIM-3 and PD-L1 [56].

### Angiogenesis

Tumor growth requires angiogenesis for nutrient supply. In the TME, certain cells, such as TAMs and CAFs secrete proangiogenic factors by induction of hypoxia [62], such as the vascular endothelial growth factor (VEGF), which can create a pro-tumor microenvironment by increasing the inhibitory function of immune cells, such as Tregs, TAMs, and MDSCs, and directly inhibit the function of cytotoxic T lymphocytes (CTLs) [63]. More importantly, following exposure to VEGF, DCs, which are required for CTL maturation, lose the ability to mature and release antigens [64]. In addition, VEGF can cause upregulation in the expression levels of the inhibitory receptors, such as TIM-3, CTLA-4, PD-1, and LAG-3 on T cells and on the expression levels of PD-L1 on tumor cells and MDSCs [65, 66]. It also directly regulates the function of epithelial cells to inhibit effector T cells [67]. VEGF promotes acquired drug resistance following treatment with ICIs by establishing the immunosuppressive TME. Therefore, it is possible to use antiangiogenic drugs to increase the antitumor effects of ICIs [68].

### EMC remodeling

Certain components of the TME can facilitate the remodeling of ECM, which promotes tumor metastasis and T cell inactivation, and enhances acquired resistance. TAMs can secrete matrix metalloproteinase (MMP) enzymes that degrade various protein components of ECM, destroy specific tissue barriers of tumor invasion, and play a key role in the process of tumor metastasis [69]. A previous study that examined human lung tumors revealed that CAFs strengthened ECM by enhancing cytokine synthesis and secretion (for instance, FGF7; hepatocyte growth factor (HGF); interleukin 6 (IL-6); PDGF; stromal cell-derived factor 1 (SDF-1)). Subsequently, they were able to hinder the transport of tumor-infiltrating lymphocytes (TILs) to tumor cells [70–72].

## Other mechanisms

In addition to the aforementioned findings, it has been reported that other mechanisms are also involved in acquired resistance.

### Radiotherapy (RT) and chemotherapy combined with immunotherapy aggravates the drug resistance phenotype

RT mainly aggravates the immunosuppressive state in the TME by promoting cytokine production. The ability of RT to activate DCs or MDSCs is dose-dependent and depends on specific induced factors. Adenosine induced by RT converts the TME from recruiting DCs and prevents their infiltration by Tregs and M2 macrophages. RT also induces VEGF production [65, 66]. RT can aggravate the hypoxic state of solid tumors, and remodel the ECM and endothelial cell architecture by promoting fibrosis, enhancing MMP activity, and upregulating FasL expression [67].

Standard chemotherapy can convert the phenotype and metabolic activity of stromal fibroblasts to those resembling CAFs. This leads to activation of the Sonic hedgehog/GLI signaling pathway [72].

### Dysbiosis plays a potential role in weakening immunotherapy

Accumulated evidence has indicated that the species and abundance of the microbiome are involved in the immune response to cancer. Gut microbiota can be divided into the two following types: 1) “Favorable” gut microbiomes, which include high diversity and abundance of *Ruminococcaceae*, *Faecalibacterium*, and *Enterococcus hirae*. These bacteria can enhance the systemic and antitumor immune responses by increasing antigen presentation and improving the effector T cell function in the periphery and the TME. 2)“unfavorable” gut microbiomes, which include low diversity and high relative abundance of *Bacteroidales*, *Staphylococcus haemolyticus*, and *Corynebacterium aurimucosum*, and limit intra-tumoral lymphoid and myeloid infiltration while weakening antigen presentation capacity [73, 74]. Large cohorts indicated that patients who received antibiotics before or soon after anti-PD1 with advanced lung, renal and urothelial cancer demonstrated reduced overall survival compared with those who did not receive antibiotics [74]. In addition, the deficiency of *Bacteroides fragilis* indicated poor immune response among patients with anti-CTLA-4 treatment [75].

## TREATMENT MEASURES AGAINST ACQUIRED RESISTANCE OF ICIS

At present, the mainstream strategies that delay or reverse the acquired resistance of ICIs include a combination with other ICIs and antitumor therapies. The combination strategies approved by FDA so far are shown in Table 1.

**Table 1:** FDA-approved combination of immunotherapy with other therapies for various cancers.

**Combination**	**Drug**	**Indication**	**Date of approval**	**Ref.**
Anti-PD-1+Anti-CTLA-4	Nivolumab+Ipilimumab	First-line treatment for intermediate- and poor-risk patients with advanced renal cell carcinoma (RCC)	April 17,2018	[76]
		Refractory dMMR–MSI-H colorectal cancer (CRC).	July 10,2018	[77]
		Unresectable malignant pleural mesothelioma	October 2, 2020	[78]
		Hepatocellular carcinoma (HCC) patients previously treated with Sorafenib	March 10,2020	[79]
		First-line treatment for patients with metastatic non-small cell lung cancer whose tumors express PD-L1(≥1%) with no epidermal growth factor receptor (EGFR) or anaplastic lymphoma kinase (ALK) genomic tumor aberrations.	May 15, 2020	[80]
Anti-PD-1+Anti-CTLA-4+chemotherapy	Nivolumab+Ipilimumab+platinum	First-line treatment for patients with metastatic or recurrent non-small cell lung cancer (NSCLC), with no epidermal growth factor receptor (EGFR) or anaplastic lymphoma kinase (ALK) genomic tumor aberrations.	May 26, 2020	[81]
Anti-PD1+chemotherapy	Pembrolizumab +Pemetrexed+Platinum	Advanced cervical cancer with disease progression	July 12,2018	[82]
		First-line treatment of patients with metastatic, non-squamous non-small cell lung cancer (NSqNSCLC), with no EGFR or ALK genomic tumor aberrations.	August 20, 2018	[83]
	Pembrolizumab+platinum	Advanced or metastatic gastric cancer, gastroesophageal junction cancer, and esophageal adenocarcinoma.	April 16, 2021	[84]
	Pembrolizumab+Platinum+fluorouracil	Head and neck squamous cell carcinoma (HNSCC)	June 10,2019	[85]
Anti-PD-L1+chemotherapy	Atezolizumab+paclitaxel protein-bound	Adults with PD-L1-positive, unresectable, locally advanced or metastatic triple-negative breast cancer (TNBC)	March 08, 2019	[86]
	Atezolizumab+ Bevacizumab+Carboplatin+Paclitaxel	First-line treatment of patients with metastatic non-squamous, non-small cell lung cancer (Nsq NSCLC) with no EGFR or ALK genomic tumor aberrations.	December 6,2018	[87]
Anti-PD-L1+Anti-VEGF	Atezolizumab+ Bevacizumab	Hepatocellular carcinoma (HCC)	May 29,2020	[88]
Anti-PD-1+Anti-VEGFR	Pembrolizumab+Axitinib	Metastatic RCC	April,2019	[89]
Anti-PD-L1+Targeted therapy	Atezolizumab+ Cobimetinib+Vemurafenib	BRAF V600 mutation-positive unresectable or metastatic melanoma.	July 30, 2020	[90]

## Combination of ICIs

The combination of ICIs, which target different enzymes, indicates potential benefits in overcoming acquired resistance in clinical trials and pre-clinical studies. Certain combination therapies have been approved by the FDA (Table 1) [91]. For example, in patients with metastatic melanoma, the median progression-free survival (PFS) was 11.5 months in the nivolumab plus ipilimumab group and 6.9 months in the nivolumab group compared with 2.9 months noted in the ipilimumab group [92]. In addition, since the expression levels of compensatory ICs were upregulated following the recurrence of tumors treated with single ICIs, such as TIM-3, LAG-3, VISTA, and TIGIT, acquired resistance was partially reversed by applying the corresponding antibodies through genetic testing. For example, the combination of anti-PD-1 with anti-TIM-3 has been used for lung cancer [93], and the combination of anti-LAG-3 and anti-PD-1 for mesothelioma and triple-negative breast cancer [94]. In mouse tumor models the co-blockade of VISTA and PD-L1 [95] was demonstrated, which improved the antitumor response [96].

## Combination of ICI treatment with chemotherapy

The combination of chemotherapy before or following ICI treatment contributes to the delay of the development of acquired resistance. The mechanism of action of the combined treatment involves enhancing tumor cell immunogenicity, the direct killing of immunosuppressive cells, and resetting the TME to favor T-cell effector functions and the formation of memory T cells [97]. For example, cyclophosphamide, platinum, and taxane can consume circulating Tregs, increase the ratio of Teffs/Tregs in the tumor, and reduce the number of MDSCs [98]. In a phase II trial, patients with advanced melanoma were locally treated by isolated limb infusion with the nitrogen mustard alkylating agent melphalan in the presence of systemic administration of ipilimumab. This combined treatment resulted in more durable PFS than ipilimumab monotherapy [99]. Additional combination strategies are shown in Table 1.

## ICIs combined with radiotherapy

RT can enhance the diversity of the T cell receptor (TCR) repertoire of intra-tumoral T cells and contribute to effector T cell activation. Local radiation therapy combined with systemic anti-CTLA-4 prolonged PFS in patients with melanoma compared with that noted (4.4 months) in patients treated with ipilimumab alone [100]. In addition, the group of anti-TIM-3 and anti-PD-L1 antibodies combined with radiotherapy further indicated a durable antitumor immune response [101]. However, the dose of radiotherapy exhibited an impact on the induction of the immune response and the effectiveness of ICIs [102].

## ICIs combined with targeted therapy

Targeted therapies may synergize with ICIs by enhancing complementary aspects of the cancer-immunity cycle, such as tumor antigenicity, T cell priming and trafficking, and infiltration into tumors. A previous study that included RCC patients indicated a median PFS of 11.7 months in patients receiving atezolizumab plus bevacizumab versus 6.1 months noted with atezolizumab monotherapy [103]. However, the toxicity caused by this combination is a major challenge [104].

## ICI combination with epigenetic drugs

Epi-drugs can modulate the sensitivity of cancer cells to anticancer therapies, including chemotherapy, radiation therapy, molecularly targeted therapy, and immunotherapy [105]. For example, HBI-8000 is a novel, orally bioavailable class I selective histone deacetylase inhibitor, which directly modifies antitumor activity by inducing apoptosis, cell cycle arrest, and resensitization to apoptotic stimuli in adult T cell lymphoma patients. This compound has been shown to augment the activity of ICIs targeting either PD-1, PD-L1 or CTLA-4, and significantly increase tumor regression [106].

## Regulation of the intestinal flora

The appropriate growth of the intestinal flora can regulate the response to ICI treatment. When using ICIs, the analysis of the taxon of the intestinal flora and the regulation of the abundance of “favorable” gut microbiomes may aid to alleviate the development of acquired resistance. Concrete measures may include fecal microbiota transplant (FMT) and orally delivered monoclonal microbiota products [107, 108]. Except for the gut microbiome, microbiota that metastasizes to the peritumoral immune microenvironment also plays a role in the response to ICIs. Pushalkar et al. demonstrated significant differences in the bacterial composition between normal pancreas and pancreatic ductal adenocarcinoma (PDAC). By using bacterial ablation, the TME can be remodeled, which is characterized by the reduction of MDSCs, the differentiation of M1 macrophages and CD4+ T cells, and the activation of CD8+ T cells. In addition, probiotics can inhibit the proliferation of tumor cells by regulating intestinal microbiota, such as by promoting the growth of short-chain fatty acid (SCFA)-producing bacteria [109].

## Tumor vaccination and oncolytic virus

The principle of tumor vaccination is to introduce tumor antigens into patients in various forms, such as tumor cells, tumor-related proteins or polypeptides, and genes expressing tumor antigens. This contributes to the enhancement of immunogenicity, by inducing patient immune responses [110]. For patients with NSCLC, combination therapies of ICIs with vaccination may be the best way to relieve acquired resistance [111].

A phase 1b clinical trial assessed the impact of oncolytic viral therapy with talimogene laherparepvec on cytotoxic T cell infiltration and the therapeutic efficacy of the anti-PD-1 antibody pembrolizumab. The data demonstrated that the combination therapy led to an increase in the number of CD8+ T cells, as well as elevated PD-L1 protein expression and IFN-γ gene expression of several cell subsets in tumors. These findings suggested that oncolytic viral therapy may improve the efficacy of anti-PD-1 therapy to reverse acquired resistance [112].

Patients with acquired resistance are treated with a single ICI, and multi-gene detection can be applied. Subsequently, the drugs are personally selected to inhibit tumor growth. For example, JAK1/2 can be detected following acquired resistance of anti-PD-1 treatment. Subsequently, the activation of type I signaling pathway or the STING stimulant can correct the mutation effects [18]. In addition, certain drug carriers have been designed to be more sophisticated and efficient, such as exosomes or nanoparticles that can be used to transport ICIs [113–115]. More convenient and reliable detection at the gene level can guide the individualized treatment of cancer patients and reduce the probability of acquired drug resistance [110].

## CONCLUSION AND EXPECTATIONS

Acquired resistance of ICIs is a disadvantage encountered in patients who were initially showing effective response to treatment. Therefore, it is important to distinguish certain patients with the poor response for ICIs, including those who exhibit primary resistance, acquired resistance, adaptive resistance, and immune escape. Subsequently, the mechanisms of acquired resistance were summarized into the three following parts: 1) The changes of tumor cell functions, including gene mutations which lead to the interruption of the IFN pathway and block the antigen presentation process, loss of tumor neoantigens, and epigenetic modifications, 2) upregulation of the expression level of ICs, such as PD1/L1, CTLA-4, LAG-3, TIM-3, TIGIT, and VISTA, 3) the interaction of various components in the TME, such as prostaglandin E2 (PGE2), arginase-1 (ARG1), adenosine, transforming growth factor β (TGF-β), interleukin (IL)-10, indoleamine2,3-dioxygenase (IDO), exosomes, matrix metalloproteinase (MMPs), and vascular endothelial growth factor (VEGF). Finally, we summarized current mainstream strategies against acquired resistance. Although neoadjuvant chemotherapy, radiotherapy, and targeted therapy may promote resistance, the combination with ICIs demonstrates the advantages of blocking or even reversing acquired resistance. In addition to ICIs, immune cell therapy, such as CAR-T (Chimeric Antigen Receptor T-Cell Immunotherapy) is a hotpot. The discovery and validation of more immune-related biomarkers can fully predict the efficiency of ICIs [9, 116]. Artificial intelligence, such as the development of machine learning and the improvement of pertinent databases, will aid the exploration of more comprehensive drug targets and more accurate personalized treatment. Immunotherapy is a rapidly developing field, which is required for the development of future therapeutic strategies.
